# Structures of Down Syndrome Kinases, DYRKs, Reveal Mechanisms of Kinase Activation and Substrate Recognition

**DOI:** 10.1016/j.str.2013.03.012

**Published:** 2013-06-04

**Authors:** Meera Soundararajan, Annette K. Roos, Pavel Savitsky, Panagis Filippakopoulos, Arminja N. Kettenbach, Jesper V. Olsen, Scott A. Gerber, Jeyanthy Eswaran, Stefan Knapp, Jonathan M. Elkins

**Affiliations:** 1 Structural Genomics Consortium, Nuffield Department of Clinical Medicine, University of Oxford, Old Road Campus, Roosevelt Drive, Oxford OX3 7DQ, UK; 2 Target Discovery Institute, Nuffield Department of Clinical Medicine, University of Oxford, Old Road Campus, Roosevelt Drive, Oxford OX3 7DQ, UK; 3 Department of Genetics, Geisel School of Medicine at Dartmouth, Lebanon, NH 03756, USA; 4 Department of Biochemistry, Geisel School of Medicine at Dartmouth, Lebanon, NH 03756, USA; 5 Department of Proteomics, Novo Nordisk Foundation Center for Protein Research, Copenhagen DK-2200, Denmark

## Abstract

Dual-specificity tyrosine-(Y)-phosphorylation-regulated kinases (DYRKs) play key roles in brain development, regulation of splicing, and apoptosis, and are potential drug targets for neurodegenerative diseases and cancer. We present crystal structures of one representative member of each DYRK subfamily: DYRK1A with an ATP-mimetic inhibitor and consensus peptide, and DYRK2 including NAPA and DH (DYRK homology) box regions. The current activation model suggests that DYRKs are Ser/Thr kinases that only autophosphorylate the second tyrosine of the activation loop YxY motif during protein translation. The structures explain the roles of this tyrosine and of the DH box in DYRK activation and provide a structural model for DYRK substrate recognition. Phosphorylation of a library of naturally occurring peptides identified substrate motifs that lack proline in the P+1 position, suggesting that DYRK1A is not a strictly proline-directed kinase. Our data also show that DYRK1A wild-type and Y321F mutant retain tyrosine autophosphorylation activity.

## Highlights

• Crystal structures of DYRK1A with ATP-mimetic inhibitor and peptide substrate
• Crystal structure of DYRK2 including NAPA and DH box region
• Determination of consensus substrate motifs for DYRK1A and DYRK2
• Observation of DYRK1A autophosphorylation on tyrosine in vitro

## Teaser

Soundararajan et al. determine structures for Down Syndrome kinases DYRK1A, with an inhibitor and consensus peptide, and DYRK2, including NAPA and DH box regions. The structures explain the roles of the activation loop tyrosine and the DH-box in DYRK activation and provide a model for DYRK substrate recognition.

## Introduction

The dual-specificity tyrosine-phosphorylation-regulated kinases (DYRKs) are an evolutionarily conserved family of kinases with five human members (DYRK1A, DYRK1B, DYRK2, DYRK3, and DYRK4). They belong to the CMGC family of serine/threonine (S/T) kinases and are categorized as class I (DYRK1A and DYRK1B) and class II (DYRK2, DYRK3, and DYRK4) DYRKs. The best-studied member of the DYRK family is DYRK1A, owing to its role in the pathology of Down syndrome and the early onset of neurodegeneration. DYRK members have been clearly shown to participate in important signaling pathways that control postembryonic neurogenesis, developmental processes, cell survival, differentiation, and death (Arron et al., 2006; Mercer et al., 2005; Tejedor et al., 1995). In addition, recent studies show DYRK1A and DYRK2 phosphorylate NFATc, countering the effect of calcium signaling and maintaining inactive NFATc (Arron et al., 2006; Gwack et al., 2006; Lee et al., 2009).

The first evidence for the key role of DYRK1A in neural proliferation and neurogenesis of the developing brain was provided by mutational analysis of the DYRK *Drosophila* ortholog minibrain (mnb), where loss-of-function mutations resulted in reduced brain size (Tejedor et al., 1995). DYRK1A is localized in the Down syndrome (DS) critical region of chromosome 21 that has been linked to the development of DS phenotypes when triplicated (Delabar et al., 1993; Sinet et al., 1994). Indeed, triplication of the DYRK1A locus in DS results in overexpression of DYRK1A in the fetal as well as adult brain and strongly implicates DYRK1A in neurodevelopmental alterations linked to some DS pathologies and disease predispositions (Dowjat et al., 2007). These links prompted studies on the role of DYRK1A in age-associated neurodegeneration and suggested DYRK1A as a target for the development of inhibitors (Mazur-Kolecka et al., 2012; Park et al., 2009). The binding modes of the inhibitors INDY and Harmine in DYRK1A have recently been published (Ogawa et al., 2010).

Apart from the well-studied DYRK1A isozyme, studies have provided evidence for the roles of DYRK1B in the development of various sarcomas (Deng et al., 2006) and in skeletal muscle differentiation (Deng et al., 2003, 2005). DYRK2 is reported to regulate key developmental and cellular processes such as neurogenesis, cell proliferation, cytokinesis, and cellular differentiation (Taira et al., 2007; Woods et al., 2001; Yoshida, 2008). Notably, DYRK2 may function in DNA damage signaling pathways, because it phosphorylates p53 at Ser46 in response to DNA damage, which induces cellular apoptosis after genotoxic stress (Taira et al., 2007). In addition, ataxia telangiectasia mutated was shown to phosphorylate nuclear DYRK2 upon DNA damage, which appeared to enable DYRK2 to protect itself from degradation that occurs due to its association with MDM2 under normal conditions (Taira et al., 2010). Emerging studies show DYRK2 has important roles in protein proteolysis, proteosomal degradation, and tumor progression (Varjosalo et al., 2008; Maddika and Chen, 2009; Taira et al., 2012). As for DYRK3 and DYRK4, their physiological functions remain poorly understood.

**Figure 1:**
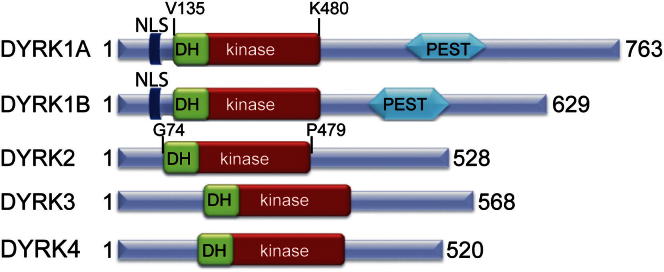
Domain Arrangement of Human DYRK Family Kinases The construct boundaries for the crystallized DYRK1A and DYRK2 proteins are indicated. NLS, nuclear localization signal; PEST, PEST domain. See also Figure S1.

All DYRKs contain a conserved catalytic kinase domain preceded by the DYRK-characteristic DYRK homology (DH) box (Figure 1A; for a sequence alignment, see Figure S1 available online). DYRKs rapidly autoactivate during folding by phosphorylation on the second tyrosine residue of the conserved activation loop YxY motif (Tyr321 of DYRK1A). This tyrosine corresponds to the secondary activation loop phosphorylation site in the TxY motif in MAPKs. It was reported based on studies with *Drosophila melanogaster* DYRKs that this phosphorylation event occurs in *cis* while DYRK is still bound to the ribosome, and subsequently DYRKs lose tyrosine phosphorylation ability and retain only S/T phosphorylation ability (Lochhead et al., 2005). For the human DYRK1A, mutation of Tyr321 or dephosphorylation did not abolish kinase activity (Adayev et al., 2007).

DYRKs were initially assumed to be proline-directed S/T kinases with specificity for proline and arginine at P+1 and P−3 positions, respectively (Himpel et al., 2000). However, further investigations revealed DYRK cellular substrates (e.g., synuclein; Kim et al., 2006) with a wide variation in phosphorylation motifs (Aranda et al., 2011).

To understand the molecular mechanism of DYRK1A activation, the roles of Tyr321 phosphorylation and regulatory elements located N-terminal to the catalytic domain, as well as substrate recognition, we determined the structure of the phosphorylated DYRK1A and DYRK2 catalytic domain and N-terminal regulatory DH box sections. The autophosphorylation behavior of DYRK1A was analyzed, and the substrate specificity of DYRK1A, DYRK1B, and DYRK2 was investigated using a novel mass spectrometry methodology (Kettenbach et al., 2012). The structure of a ternary substrate complex of DYRK1A, the ATP-mimetic inhibitor DJM2005, and a consensus substrate peptide (RARPGT^*^PALRE) reveals how DYRK1A recognizes substrates and provides a model for the structure-based design of selective DYRK inhibitors.

## Results

### Structures of the DYRK1A and DYRK2 Catalytic Domain and DH Box

**Figure 2:**
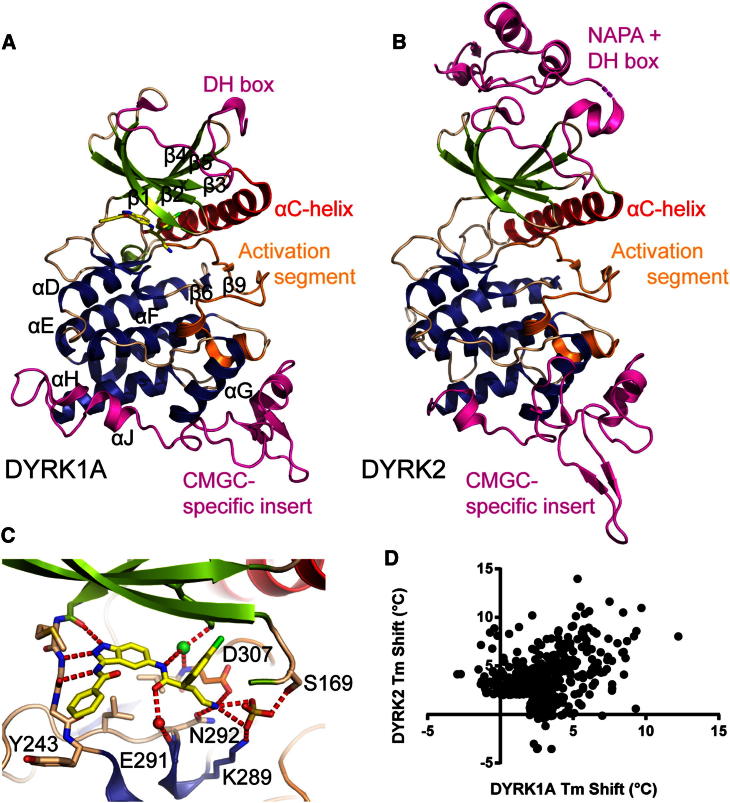
Structures of DYRK1A and DYRK2 (A) Structure of DYRK1A kinase domain and DYRK homology box with the inhibitor DJM2005 bound in the ATP binding site. The DH box and CMGC-specific inset are shown in magenta and the activation segment in orange. (B) Similar view of DYRK2 showing the NAPA region and DH box in magenta. (C) Active site of DYRK1A with inhibitor DJM2005 bound. Part of strand β1 has been removed for clarity. The inhibitor is colored yellow, the protein is colored as in (A), and hydrogen bonds are shown as dashed red lines. See also Figure S3. (D) Correlation of the binding of various kinase inhibitors (measured by ΔT_m_) to DYRK1A and DYRK2 showing that whereas some inhibitors bind both proteins, the active site differences allow for DYRK1A- or DYRK2-specific inhibitors.

The crystal structures of DYRK1A and DYRK2 comprising the catalytic kinase domain and DH box were determined. The DYRK1A structure was determined from a construct expressing residues 127–485 of human DYRK1A (National Center for Biotechnology Information [NCBI] genInfo identifier [gi] number 18765758). Residues Val135–Lys480 comprising the DH box and kinase domain were resolved in the electron density. The structure was determined in complex with the ATP-competitive inhibitor (*S*)-N-(5-(4-amino-2-(3-chlorophenyl)butanamido)-1H-indazol-3-yl)benzamide (DJM2005) at 2.40 Å resolution (Figure 2A; Table 1). The inhibitor DJM2005 was kindly provided by the laboratory of Kevan Shokat; the chemical structure is shown in Figure S2.

**Table 1:** Data Collection and Refinement Statistics

	DYRK1A-Inhibitor^a^	DYRK1A-Inhibitor^a^-Peptide	DYRK2
PDB ID code	2VX3	2WO6	3K2L
Crystallization conditions	4% (v/v) PEG 300, 0.1 M Li_2_SO_4_, 0.1 M Tris, pH 8.5	0.2 M sodium formate, 20% (w/v) PEG 3350, 10% ethylene glycol	1.26 M (NH_4_)_2_SO_4_, 0.2 M Li_2_SO_4_, 0.1 M Tris, pH 8.5
Space group	*C*2	*P*6_5_	*P*4_2_2_1_2
No. of molecules in the asymmetric unit	4	2	1
Unit cell dimensions			
*a*, *b*, *c* (Å)	264.2, 65.1, 140.3	168.4, 168.4, 62.4	84.3, 84.3, 148.5
α, β, γ (°)	90.0, 115.44, 90.0	90.0, 90.0, 120.0	90.0, 90.0, 90.0

**Data Collection**			

Beamline	SLS X10SA	Diamond I02	Diamond I03
Resolution range (Å)^b^	27.24–2.40 (2.53–2.40)	55.13–2.50 (2.64–2.50)	42.19–2.36 (2.49–2.36)
Unique observations^b^	85,770 (12,458)	35,283 (5,093)	22,801 (3,275)
Average multiplicity^b^	3.4 (3.2)	7.5 (6.9)	6.2 (6.4)
Completeness (%)^b^	99.9 (99.9)	100.0 (100.0)	99.8 (100.0)
R_merge_^b^	0.10 (0.82)	0.18 (0.57)	0.08 (0.90)
Mean (*I*)/σ(*I*)^b^	9.5 (1.9)	10.8 (3.7)	12.2 (2.1)

**Refinement**			

Resolution range (Å)	26.00–2.40	40.00–2.50	42.19–2.36
R value, R_free_	0.19, 0.23	0.19, 0.23	0.23, 0.29
Mean protein B values (Å^2^)	53	23.1^c^	33.8^c^
Mean ligand B values (Å^2^)	46 (inhibitor)	34 (inhibitor)	
		71 (peptide)	
Rmsd from ideal bond length (Å)	0.014	0.014	0.014
Rmsd from ideal bond angle (°)	1.52	1.53	1.60
Ramachandran outliers (%) Most favored (%)	0.15	0.0	0.0
	96.1	96.3	95.0

a The inhibitor structure is shown in Figure S2.

b Values within parentheses refer to the highest resolution shell.

c Residual after TLS parameterization.

The DYRK2 structure was determined from a construct expressing residues 74–479 of human DYRK2 (NCBI gi number 4503427). Residues Gly74–Pro470 comprising NAPA1 (*N*-terminal *a*uto*p*hosphorylation *a*ccessory 1), NAPA2, DH box, and kinase domain were resolved in the electron density, as well as part of the N-terminal purification tag. The structure was determined in the absence of inhibitor (apo form) at 2.36 Å resolution (Figure 2B; Table 1).

For both DYRK1A and DYRK2 the entire catalytic domain was well ordered, including a long hairpin-like structure for the N-terminal DH box and an active kinase conformation with a fully ordered activation segment (Figure 2). Mass spectrometry showed that the purified DYRKs were heterogeneously phosphorylated in solution (data not shown). However, the electron density maps only showed clear evidence of phosphorylation of DYRK1A at the second tyrosine of the dual-phosphorylation motif YxY (Tyr321) and double phosphorylation of DYRK2 at Ser159 of the glycine-rich loop and Tyr309 of the activation loop. The other phosphorylation sites might either have had low occupancy or were located in unstructured regions of the protein.

The DYRK1A and DYRK2 structures superimpose with a root-mean-square deviation (rmsd) of 1.03 Å over 297 Cα atoms (using chain A of the DYRK1A structure). In DYRK1A, the ATP-mimetic inhibitor DJM2005 binds to the ATP binding site, forming three hydrogen bonds with the hinge backbone and an additional two hydrogen bonds from the inhibitor’s primary amine with the side chains of Asn292 and the DFG motif aspartate Asp307 (Figure 2C). There is also an electrostatic interaction via an ion (modeled as chloride) linking an inhibitor amide nitrogen to the backbone nitrogen of Asp307 from the DFG motif, and hydrogen bonding via a water molecule to the backbone carbonyl of Glu291. There are various favorable hydrophobic interactions with DYRK1A active site residues, including at the entrance to the ATP site, where the side chain of Tyr243 packs against the inhibitor’s phenyl ring. All of the DYRK1A residues involved in hydrogen bonding to the inhibitor are conserved in DYRK2 (Figure S3); there are, however, some potential differences in the hydrophobic interactions, such as the replacement of Tyr243 with Met233 in DYRK2 as well as differences at the back of the pocket and the hydrophobic residue preceding the DFG motif. Analysis of changes in DYRK1A and DYRK2 temperature shift values (ΔT_m_) in the presence of a set of potential kinase inhibitors showed only weak correlation, and therefore that it is possible to have DYRK1A- or DYRK2-specific inhibitors, as shown by some of the inhibitors screened that give changes in T_m_ with only DYRK1A or only DYRK2 (Figure 2D).

Interestingly, the inhibitor’s primary amine also interacts with a sulfate molecule from the DYRK1A crystallization buffer that is found in a similar location as an autophosphorylated serine residue in DYRK2 (pS159; Figure S3). This sulfate is also bound by the side chains of Asp307 of the DFG motif, Ser169 of the glycine-rich loop, and Lys289 of the catalytic loop, and is in a similar position as that of a hydrolyzed γ-phosphate from ATP bound to PKA (Protein Data Bank [PDB] ID code 1RDQ; Yang et al., 2004) or a bound phosphate in the structure of Haspin with a 5-iodotubercidin ligand (PDB ID code 3IQ7; Eswaran et al., 2009). Addition of negatively charged groups to inhibitors to exploit this conserved binding pocket may help inhibitor design for some of these kinases.

The C-terminal lobe reveals several unique features that define the DYRK family. The MAP kinase characteristic insertion observed in the C lobe of DYRKs (Figure 2) is extended in comparison with other CMGC family members such as CLK1, CLK3 (Bullock et al., 2009), GSK3β (Dajani et al., 2001), or MAPKs (Canagarajah et al., 1997). In DYRK1A, this insert forms an elaborate subdomain of 40 residues comprising two short helices followed by an antiparallel β sheet that is conserved in vertebrate DYRK1 family members but not in *Drosophila* mnb. In DYRK2, this insert also forms a distinctive subdomain with two short helices and three short antiparallel β sheets. Along with the structural divergence between DYRK1A and DYRK2 in this insertion, this region is also the place of greatest divergence among the other DYRK family members.

### Regulatory Role of the N-Terminal Region

Deletion of the entire region of DYRK1A N-terminal to the kinase domain (1–148) has been shown to decrease catalytic activity (Himpel et al., 2001). In *Drosophila*, the DH box was required for phosphorylation of SNR1 by DYRK2 but not by DYRK1 (Kinstrie et al., 2006). With *Drosophila* DYRKs, the NAPA regions are required for the transient intramolecular tyrosine kinase activity of DYRKs (Kinstrie et al., 2010) and are conserved across a wide range of eukaryotes, including for *Trypanosoma brucei* DYRK2, where the NAPA1 and NAPA2 regions are required for tyrosine autophosphorylation (Han et al., 2012). In the following analysis, the DH boxes and NAPA regions are those defined by Kinstrie et al. (2010).

**Figure 3:**
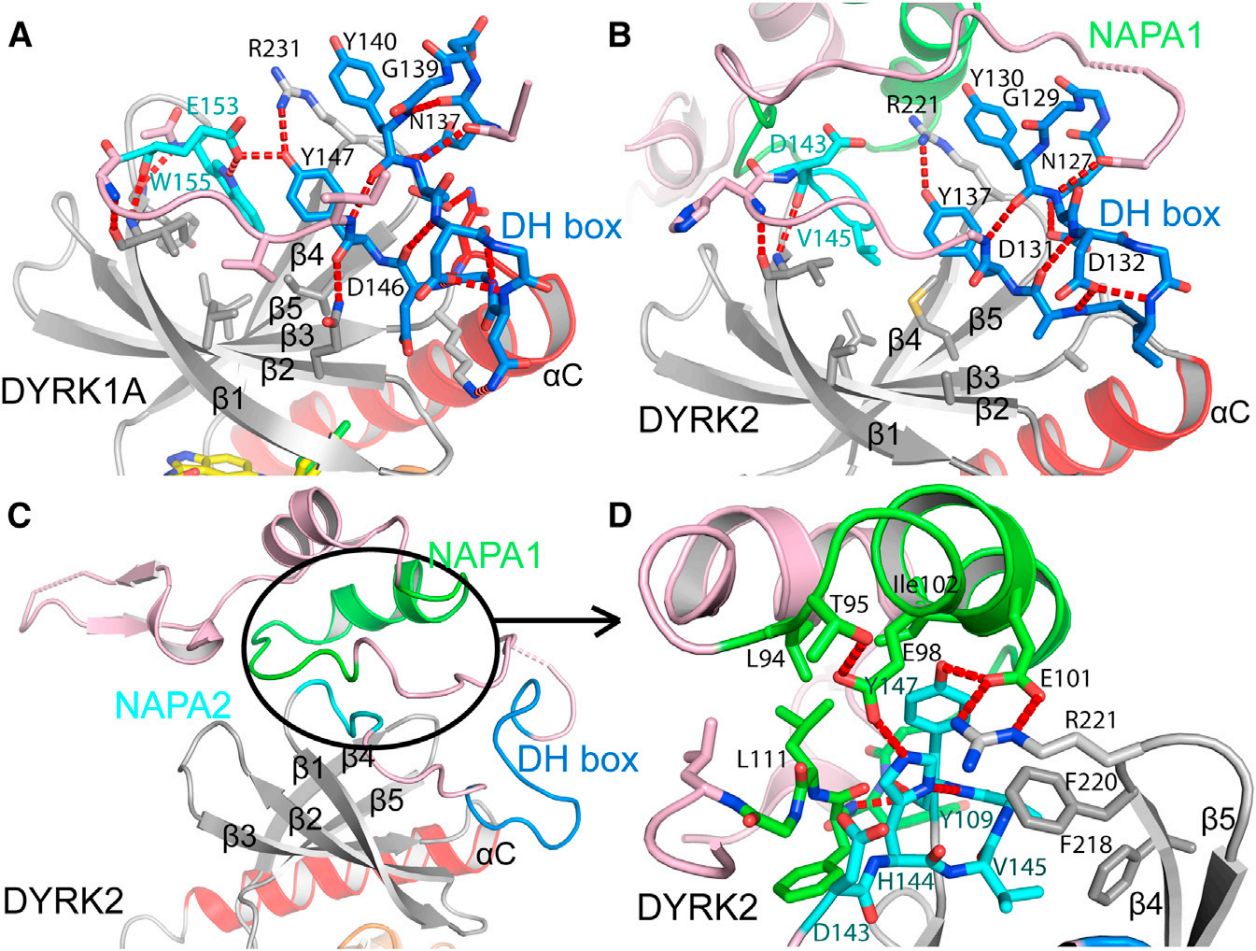
The N-Terminal NAPA and DH Box Regions The DH box region is in dark blue, the NAPA1 region for DYRK2 is in green, and the NAPA2 regions for DYRK1A and DYRK2 are in cyan. Residues in these motifs that are highly conserved across DYRK kinases from different species (Kinstrie et al., 2010) are labeled. (A) DH box region of DYRK1A. (B) DH box region of DYRK2. (C) Overview of the N terminus of DYRK2 showing NAPA and DH box motifs. (D) NAPA1 and NAPA2 regions of DYRK2 showing their folded, assembled state. See also Figure S4.

In both DYRK1A and DYRK2, the N-terminal region containing the DH box is positioned on top of the N-terminal lobe of the kinase domain and forms a large network of interactions with all five strands of the N lobe β sheet, providing considerable stabilization (Figures 3A and 3B). The most highly conserved residues in the DH box are those essential for stabilization of its folded state, in particular the two central tyrosines, Tyr140 and Tyr147, in DYRK1A (Figure 3A). This compact folded DH box appears essential for the formation of tertiary structure in the remainder of the N terminus, especially for class II DYRKs, which have NAPA1 and NAPA2 regions (Figure 3C). Although many of the DH box interactions are conserved between DYRK1A and DYRK2, we observed more hydrogen-bonding interactions in the DYRK1A structure. In particular, in DYRK1A, the central tyrosine, Tyr147, interacts with the DYRK1A equivalent of the NAPA2 region, Glu153 and Trp155. These residues are not present in the standard NAPA2 region of DYRK2, and DYRK2 does not have an equivalent interaction between the DH box and NAPA2 regions (Figure 3B). Recent evidence suggests phosphorylation of DYRK1A at Tyr145 and Tyr147 may have important regulatory roles (Kida et al., 2011). Tyr145 is solvent exposed, but phosphorylation of Tyr147 would change its interactions significantly, although it is not possible to predict whether this would be favorable or unfavorable, because pTyr147 could maintain interactions with Arg231 and replace the interactions of Glu153.

As well as the stabilization provided by the N-terminal region, the 11 residues of the DH box itself interact with the loop linking αC with β3, providing stabilization to an “αC-in” active kinase conformation by fixing the N-terminal end of αC in position and so preventing αC from moving outward, as sometimes seen in inactive kinase structures (Figure 3A). Interestingly, the interaction appears not to be charge dependent, unlike for the interaction of the N-terminal hairpin of CLK3 (Bullock et al., 2009) with the kinase N-terminal lobe (Figure S4).

The DYRK2 NAPA1 and NAPA2 regions, which are separated in sequence, fold together into a small domain that stabilizes the N-terminal lobe of the kinase domain (Figures 3C and 3D). The larger NAPA1 region folds around the five residues of the NAPA2 region. As with the DH box, the most highly conserved residues (marked in Figure 3D) are those forming the core of this folded subdomain, in particular His144 and Tyr147 from NAPA2. It is notable that the residues on the end of strand β4 (DYRK2: Phe218, Phe220, Arg221), which interact with the NAPA2 region (Figure 3D), are conserved across all human DYRKs (Figure S1). For DYRK1A, an early folded intermediate is implicated in enabling transient Tyr autophosphorylation in *cis* (Lochhead et al., 2005). The presence of small N-terminal domains (DH/NAPA1/NAPA2) capable of folding independently and that stabilize the kinase domain may explain how during translation a stabilized and catalytically active conformation can be achieved before translation is complete.

### The DYRK Activation Segment Is Stabilized by Tyr Phosphorylation

**Figure 4:**
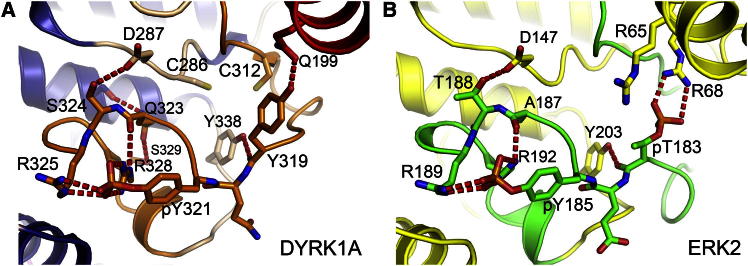
Activation Loop and Active State Stabilization Comparison of the activation segment arrangements of (A) DYRK1A, activation segment in orange, and (B) dual-phosphorylated ERK2, activation segment in green. The ERK2 structure is from PDB ID code 2ERK. Both DYRK1A and ERK2 have a completely ordered activation loop and glycine-rich loop, and active αC conformations. The activation loop in dual-phosphorylated active ERK2 forms an extensive hydrogen-bonding network around pT183. Phosphorylated Y185 is also stabilized through an extensive interaction network that is similar to the pY321 network formed by DYRK1A.

The structures of DYRK1A and DYRK2 both show a completely ordered activation segment in a similar conformation (Figure 2). The second tyrosine of the YxY dual-phosphorylation motif (DYRK1A: Tyr321; DYRK2A: Tyr309) is the main mediator of a network of interactions that stabilize the active conformation, including with Arg325 and Arg328 (numbering for DYRK1A) that precede the APE motif, and with the backbone carbonyl of the catalytically important Gln323 (Figure 4A).

Related CMGC kinases such as ERK2 have a TxY motif, and phosphorylation of the first residue of this motif, the threonine, allows formation of salt bridges with the arginine of the usually conserved activation segment HRD motif, neutralizing its charge (Canagarajah et al., 1997; Dajani et al., 2001). However, in all DYRK kinases, the HRD arginine is replaced by a cysteine, suggesting that phosphorylation of the primary phosphorylation site is not required for activity. In both DYRKs, the HCD motif cysteine (DYRK1A: Cys286; DYRK2: Cys274) is within range of potential disulfide-bond formation with a cysteine at the beginning of the activation loop (DYRK1A: Cys312, 4.4 Å distant; DYRK2: Cys300, 4.2 Å distant), raising the possibility that DYRK kinase activity might be regulated by the cellular redox state (Figure 4A). No disulfide bonds were observed in the crystallized proteins, which were prepared under reducing conditions.

Comparison with the diphosphorylated ERK2 structure (PDB ID code 2ERK) reveals only small differences in the overall activation loop conformation, mainly due to sequence and loop length variations (Figures 4A and 4B). However, the positions of the TxY/YxY motifs are well conserved. The primary phosphorylation site pT183 in ERK2 links αC (Arg68) with the activation segment and with the catalytic loop, the HRD motif. In DYRKs, the first tyrosine residue (DYRK1A: Tyr319; DYRK2A: Tyr307) forms a hydrogen bond with Gln199 from αC. Therefore, there is an interaction with αC that is independent of phosphorylation; nevertheless, the direct contribution of DYRK1A Tyr319 toward catalytic activity and tyrosine autophosphorylation has been found to be negligible (Himpel et al., 2001). The phosphate moiety of the second YxY motif tyrosine is therefore the major activation loop phosphorylation site for DYRKs; it forms similar interactions as those of the secondary phosphorylation site of ERK2 at Tyr185.

### DYRK1A Autophosphorylates Serine and Threonine as Well as Tyrosine In Vitro

**Figure 5:**
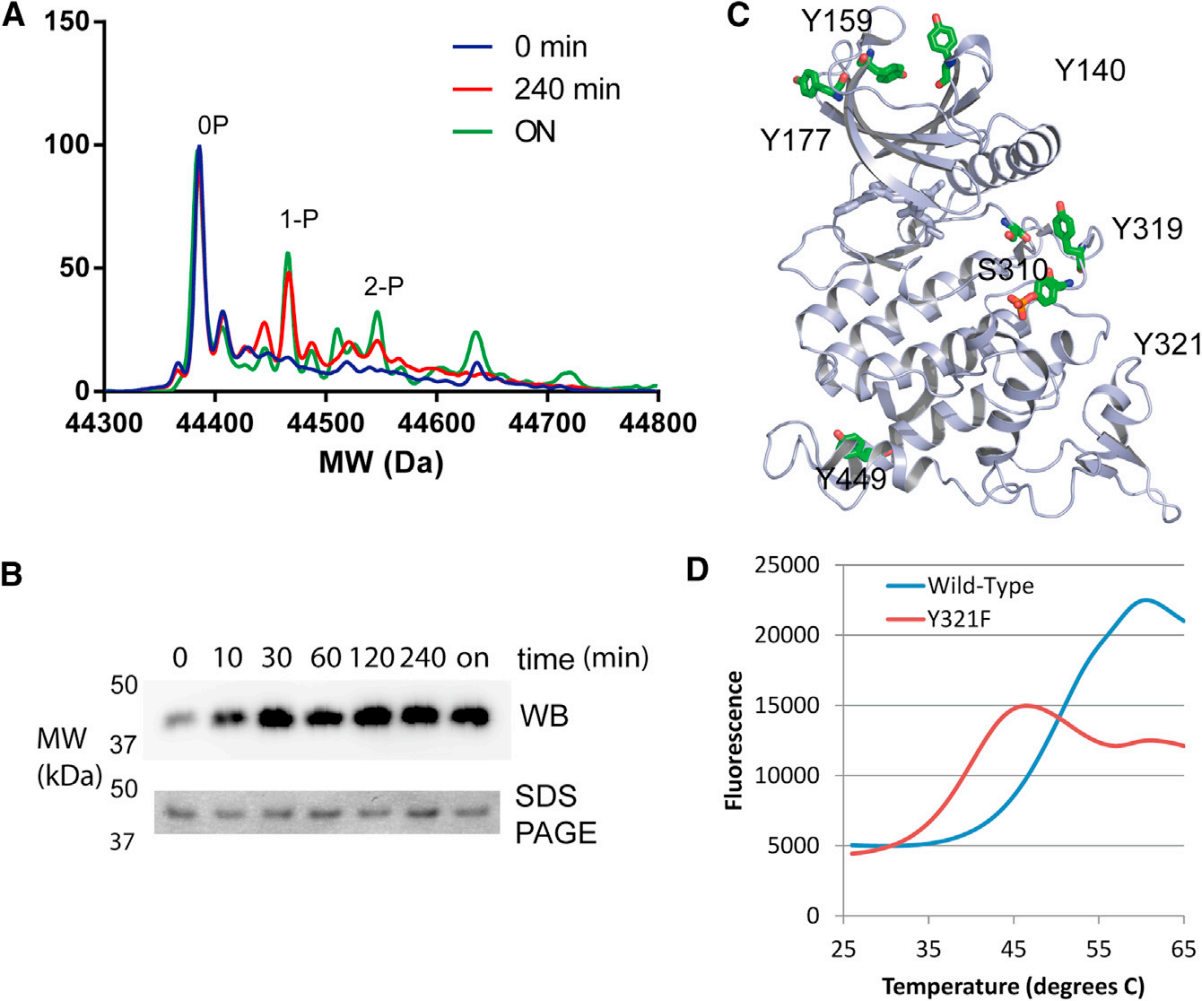
Phosphomapping and Autophosphorylation (A) Autophosphorylation kinetics of DYRK1A Y321F showing electrospray ionization-MS spectra recorded after 0 hr, 4 hr, and overnight. (B) Phosphorylation capability of DYRK1A Y321F mutant. The top panel shows a western blot of DYRK1A Y321F autophosphorylation probed by anti-phosphotyrosine antibody after the reaction times indicated. The bottom panel shows a quantitative control with equal amounts of sample run on the gel. Related to Figure S6. (C) The autophosphorylation sites mapped for wild-type DYRK1A are shown on the structure as green sticks. (D) Thermal unfolding of DYRK1A wild-type and Y321F mutant. See also Figure S6.

The intact mass spectra of purified DYRK1A and DYRK2 clearly indicated multiple phosphorylation states. We coexpressed DYRK1A with λ-phosphatase in bacteria, yielding DYRK1A singly phosphorylated at Tyr321. This protein was subjected to autophosphorylation in vitro, generating up to three additional phosphorylation sites after the reaction ran to completion, as measured by mass spectrometry. The resulting sites were mapped by liquid chromatography-tandem mass spectrometry (LC-MS/MS) (Table 2; Figure S5); sites were identified at Tyr140 and Tyr159 in the DH box region, Tyr177 in β2 of the N-terminal lobe, Ser310 immediately C-terminal of the DFG motif, Tyr319 of the YxY motif, and Tyr449 located near the C terminus of the molecule (Figure 5C). To rule out that tyrosine autophosphorylation was due to the short construct used for crystallization, we generated additional constructs of DYRK1A 1–485 and 37–485 and again looked for autophosphorylation sites. LC-MS/MS confirmed Tyr autophosphorylation on Tyr111, which was not present in the shorter constructs. The mapped sites demonstrate the capability of DYRK1A to autophosphorylate on tyrosine after the formation of mature, folded protein in vitro, which is contradictory to previous data based on experiments with *D. melanogaster* DYRKs, which reported tyrosine phosphorylation as a one-time-only event during translation (Lochhead et al., 2005). However, these data agree with reports of weak Tyr phosphorylation observed by Adayev et al. (2007) using a specific pTyr antibody. The distant location of many sites from the active site also suggests that most of these phosphorylations were carried out in *trans* rather than in *cis*. Although all the identified phosphorylation sites are conserved within the DYRK family and also across various species, the Tyr phosphorylation is weak, and any physiological significance of phosphorylation at these phosphorylation sites is yet to be established. Interestingly, phosphorylation on Tyr145 and Tyr147 has recently been identified as a modification that determines nuclear localization of DYRK1A in neurons (Kida et al., 2011).

**Table 2:** Phosphopeptides Identified following Autophosphorylation of DYRK1A

Sequence	Residue Range
A**pY**DRVEQEWVAIK	176–188
IVDFG**pS**SCQLGQR	305–317
I**pY**Q**pY**IQSR	318–325
IYQ**pY**IQSR	318–325
LPDG**pT**WNLK	398–406
RAGESGHTVAD**pY**LK	438–451
VYNDG**pY**DDDNYDYIVK	135–150
WM(ox)DR**pY**EIDSLIGK	155–167
WMDR**pY**EIDSLIGK	155–167
HINEV**pY**YAK^a^	106–114

Related to Figure S5.

a Only observed for DYRK1A 1–485 or 37–485, not for DYRK1A 127–485.

To analyze the importance of Tyr321 phosphorylation for activity, we also measured autophosphorylation kinetics for a DYRK1A Y321F mutant. This mutant was not phosphorylated after coexpression with λ-phosphatase, but under the same in vitro reaction conditions we observed autophosphorylation activity (Figures 5A and 5B). To verify the activity of our DYRK1A 126–485 constructs and the suitability of the consensus peptide used for cocrystallization (see below), we measured activity of the 126–485 wild-type and Y321F mutant in an in vitro assay (Figure S6). Y321F had 72% of wild-type activity against the consensus peptide, in general agreement with previous activity measurements on other DYRK1A constructs (Himpel et al., 2001). To assess the importance of pTyr321 for DYRK1A stability, we measured ΔT_m_ data for DYRK1A 126-485 wild-type and Y321F; the Y321F mutant melted at an approximately 12°C lower temperature compared to wild-type (Figure 5D).

### The Phosphorylation Substrate Recognition Motifs of DYRKs

**Figure 6:**
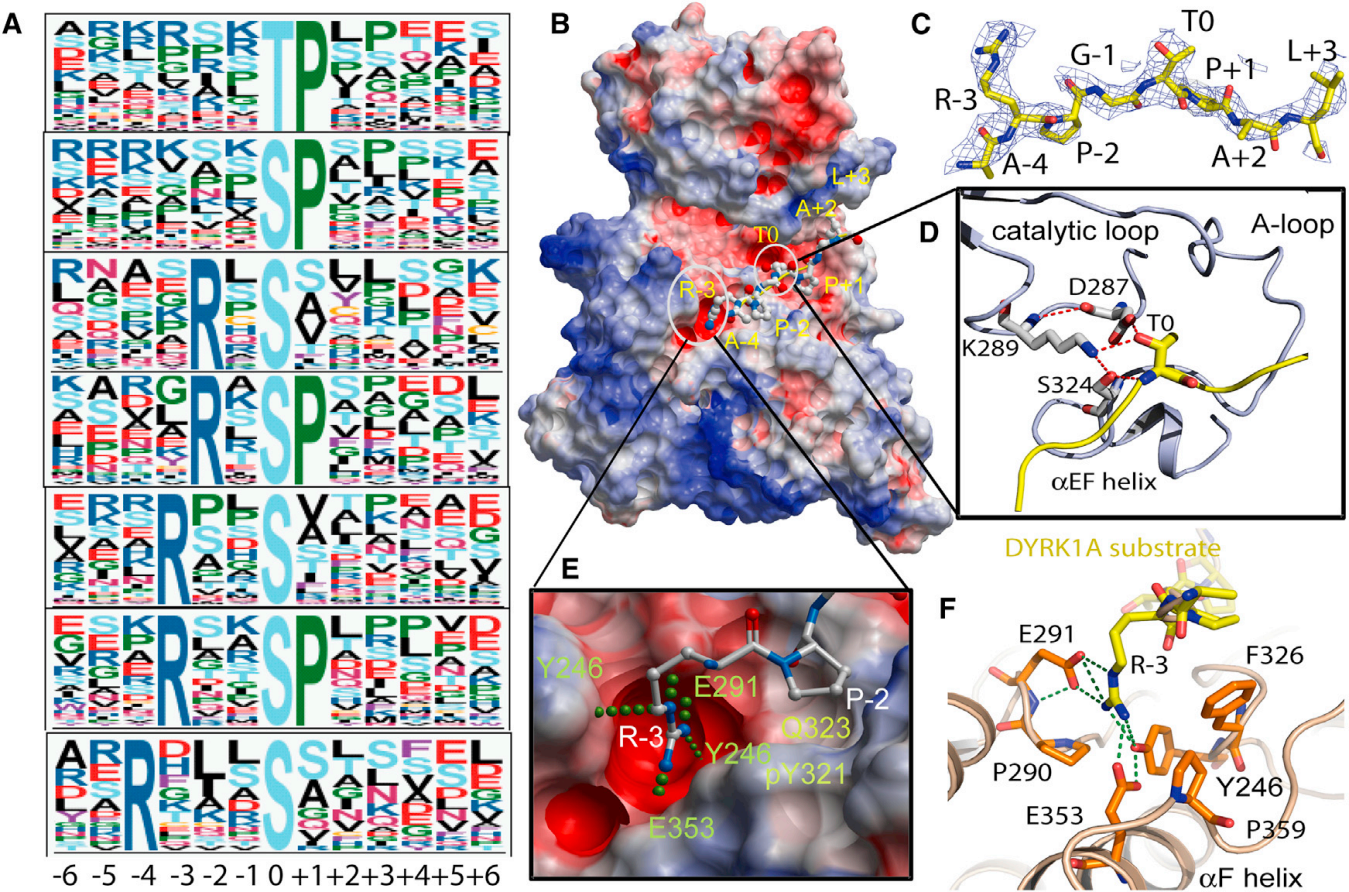
Substrate Binding of DYRK1A (A) Representative panels of DYRK1A substrate specificity defining peptide residues identified using in vivo isolation. Each panel represents a separate clustering of peptide sequences, with the most commonly observed residues at each position at the top of each letter stack. Within each clustering, the fraction of the height occupied by each residue represents its predominance at that position. (B) Ternary complex of DYRK1A substrate peptide and inhibitor DJM2005 bound to DYRK1A. The substrate peptide is shown bound between the two lobes of the kinase in the binding cleft extending from the ATP site toward helix αC. The DH box and kinase domain of DYRK1A are shown as an electrostatic surface representation, with the substrate peptide in white balls and sticks and residues of substrates labeled with reference to the phosphoacceptor residue (threonine T0). (C) Electron density map of the DYRK1A substrate peptide for the 8 out of 11 residues for which the density was visible in the structure, numbered with respect to phosphoacceptor threonine T0. (D) Close-up view of the atomic arrangement around the phosphoacceptor residue threonine T0. (E) Close-up view of the arginine binding pocket of DYRK1A, where the arginine at position −3 of the substrate binds to negatively charged residues of the C lobe (red-colored surface) and forms an extensive bonding network with residues from the activation loop, αD and αF. (F) Stick representation similar to (E). See also Figure S7.

Initially, DYRKs were considered to be proline-directed kinases with a similar recognition motif as ERKs. However, subsequent biochemical studies identified substrates with a variety of recognition sequences. We employed an in vitro kinase substrate screening method using naturally occurring substrates from HeLa cells (Kettenbach et al., 2012) to identify the substrate recognition motifs for class I and II DYRKs, DYRK1A and DYRK2. The results showed that DYRK1A phosphorylates substrates exclusively on serine or threonine residues, on peptides that have smaller hydrophobic residues at the P+1 position (Figure 6A). Another notable preference observed was arginine at P−2 to P−4, positions poised for occupying the C lobe electronegative pocket. In the case of DYRK2, proline is strongly preferred at P+1, recognizing S/TP motifs and S/TPxP motifs, but the arginine preference at P−2 to P−4 is not as strong as with DYRK1A (Figure S7). Arginine at P−3 has been previously shown to be more favorable than at P−2 on a small selection of artificial peptides with DYRK2 and DYRK3 (Campbell and Proud, 2002). On a larger set of nonendogenous peptides, preference for P−3 arginine was shown to be a feature of DYRK1A but not DYRK2 or DYRK4 (Papadopoulos et al., 2011).

The substrate specificities identified from HeLa cell extracts correlate well with previous studies that have identified DYRK1A substrates such as Tau (Ryoo et al., 2007; Liu et al., 2008), amphiphysin (Murakami et al., 2006), and caspase 9 (Seifert et al., 2008) that conform to the above definition (proline at P+1 position and arginine at P− positions), whereas other studies report substrates such as spliceosomal protein SF3b1 (de Graaf et al., 2006) that contain proline in the P+1 position but not basophilic residues at P− positions. Furthermore, DYRK1A substrates α-synuclein (Kim et al., 2006) and p53 (Park et al., 2010) do not contain either of these substrate specificity determinants, indicating the flexibility of DYRK1A in substrate recognition.

### Ternary Complex Structure of DYRK1A Comprising Consensus Substrate Peptide

To further explore the substrate recognition of DYRKs, we determined the crystal structure of the ternary complex of DYRK1A with the consensus substrate peptide RARPT^*^PALRE and DJM2005 (Figure 6B). Although several consensus peptides were used in cocrystallization experiments, crystals were only obtained with this substrate peptide. Electron density for 8 of the 11 peptide amino acids was visible, from P−4 Ala to P+3 Leu. The peptide binds in an extended conformation in the cleft formed by the activation segment and catalytic loop. There were no major protein conformational changes upon peptide binding. The hydroxyl group of the P0 threonine forms hydrogen bonds with the catalytic loop aspartate (Asp287) and the highly conserved Lys289 of the catalytic loop flanking region and Ser324 of the activation loop (Figure 6D). The P+2 leucine and P+3 alanine bind against a hydrophobic pocket formed by Phe196 from αC and Phe170 from the glycine-rich loop, explaining the preference for hydrophobic residues at these substrate positions. The P+1 proline was bound adjacent to the catalytically important Gln323 (Wiechmann et al., 2003) and also against the aromatic ring of pTyr321 (Figure 6E). The small size and lack of charge in this pocket explains why only substrates with small aliphatic residues at P+1 were acceptable. The P−3 arginine was bound in a negatively charged pocket formed by Glu291, Tyr327, Tyr246, and Glu353 (Figures 6E and 6F). Interestingly, it faces toward the activation segment in the C lobe, not the glycine-rich loop, but stabilizes the αD helix through the interactions with Tyr246 on αD. However, the binding pocket is extended and so it may also accommodate substrate arginines located in the −2 and −4 positions, again fitting with the observed DYRK1A substrate profile.

## Discussion

It has been established that DYRKs autoactivate shortly after translation through autophosphorylation of the YxpY motif of the activation segment (Becker et al., 1998; Becker and Joost, 1999; Lochhead et al., 2005). The DYRK structures revealed that phosphorylation of Tyr321 stabilizes the activation segment in a similar way to that observed for phosphorylated ERK2 (Canagarajah et al., 1997). The rapid autoactivation of DYRK kinases immediately after translation raises the question of how DYRK activity is regulated. Mouse models and truncations of DYRK1A in patients with microcephaly demonstrated that deregulation of DYRK1A activity has severe phenotypic consequences (Fotaki et al., 2002; Møller et al., 2008). Several reports point to transcriptional regulation, and the presence of PEST sequences (Figure 1) suggests a quick turnover (Maenz et al., 2008; Arron et al., 2006).

The current model suggests that Tyr321 phosphorylation is a “one-time-only” event that happens during maturation of DYRK1A while bound to the ribosome. For class II DYRKs (DYRK2, 3, and 4), this process requires the N-terminal NAPA regions, which are absent/modified in class I DYRKs (DYRK1A and 1B). The DYRK2 structure shows how the DH box and NAPA regions bind across the N-terminal lobe of the kinase domain, via significant conserved interactions at the kinase domain loops between β3 and αC and between β4 and β5. Thus, the DH box and NAPA regions come together to form a domain that can fold before translation of the kinase domain is complete and assist in the folding of the kinase domain. Presumably, this enables a partially folded intermediate with the previously observed transient tyrosine kinase capability.

However, our data, with human enzymes as opposed to the *D. melanogaster* enzymes used previously (Lochhead et al., 2005), also showed that tyrosine autophosphorylation can occur weakly in vitro and is not restricted to the Tyr321 site (DYRK1A) and that this activity is independent of Tyr321 phosphorylation, although a possible caveat is that the constructs used were the same as those used for crystallization, and not the full-length protein. In vitro autophosphorylation at Tyr111 has been reported previously (Himpel et al., 2001), but mutation of this residue was shown to have no influence on catalytic activity. Tyr111 is located N-terminal to the DH box and was not included in the construct used for the crystal structure. The importance of phosphorylation of Tyr321 and Tyr319 for activity has been reported by several groups (Himpel et al., 2001; Lochhead et al., 2005). These studies showed that Tyr321 is the main phosphorylation site observed in recombinant protein expressed in both bacteria and eukaryotic cells. Mutation Y321F dramatically reduces catalytic activity, whereas Y319F does not alter activity (Adayev et al., 2007; Wiechmann et al., 2003). Some other mutations (e.g., double mutants Y319Q/Y321Q and Y319H/Y321H) also retain considerable activity (Adayev et al., 2007). The DYRK1A structure provides explanations for these observations, as the mutations to polar side chains would still allow the activation segment to retain favorable polar interactions with residues that coordinate pTyr321 (Arg325, Arg328, Glu366). By analogy, the activity of nonphosphorylated Tyr321 (Adayev et al., 2007) is also explained by the continued ability of Tyr321 to retain some favorable polar interactions that would stabilize an active conformation of the activation loop.

Substrate peptide profiling did not reveal any tyrosine phosphorylation, suggesting that tyrosine kinase activity is limited to autophosphorylation events. Comparison of the DYRK1A substrate complex with substrate-bound tyrosine kinase structures shows little similarity, and the substrate binding site in DYRK1A has the typical appearance of those in S/T kinases. Kinase autophosphorylation has been shown to frequently target nonconsensus sequences (Pike et al., 2008; Oliver et al., 2007). Moreover, our data revealed that DYRK1A S/T phosphorylation activity is not stringently proline directed and that substrates with small hydrophobic residues such as valine or alanine in the P+1 position can be recognized.

Finally, as well as explaining the observed substrate specificities, the presented structures will serve as a model for the development of more potent and selective ligands that might find application in the treatment of neurodegenerative diseases.

## Experimental Procedures

### Cloning

DNA for DYRK1A residues 127–485 (NCBI gi number 18765758) or DYRK2A residues 74–479 (NCBI gi number 4503427) was PCR amplified and subcloned into a pET-based vector carrying kanamycin resistance, pNIC28-Bsa4 (GenBank accession number EF198106), using ligation-independent cloning. The resulting plasmids expressed the kinase domains of DYRK1A or DYRK2, with an N-terminal hexahistidine tag and tobacco etch virus protease tag cleavage site (extension MHHHHHHSSGVDLGTENLYFQ^*^SM-). DYRK1A 1–485 and 37–485 constructs were generated similarly. DYRK1A 127–485 Y321F and K188R mutants were generated by the overlapping PCR product method.

### Expression and Purification

Constructs were used to express protein in *Escherichia coli* BL21 (DE3) cells, and protein was purified using standard methods (see Supplemental Experimental Procedures).

### Crystallization and Data Collection

All crystals were obtained using the sitting-drop vapor-diffusion method at 4°C. Data collection statistics and crystallization conditions can be found in Table 1. More detail is available in Supplemental Experimental Procedures.

### Structure Determination

All diffraction data were indexed and integrated using MOSFLM (Leslie and Powell, 2007) and scaled using SCALA (Evans, 2006). All models were refined with REFMAC5 (Murshudov et al., 2011).

The DYRK1A structure was solved by molecular replacement using Phaser (McCoy et al., 2007) and a search ensemble of the coordinates from two CLK kinases (PDB ID codes 2EXE and 1Z57). Four molecules were present in the asymmetric unit and, after NCS averaging and density modification in dm (Cowtan, 1994), the resulting phases could be utilized in ARP/wARP (Langer et al., 2008) to autobuild the main parts of one of the molecules in the asymmetric unit. After further model building in Coot (Emsley et al., 2010), this molecule was used to generate the other three molecules for restrained refinement with tight NCS restraints. Rebuilding and refinement (including TLS parameters) resulted in the final model.

The DYRK1A peptide complex and the DYRK2 structure were both solved by molecular replacement using Phaser, with the structure of the inhibitor-bound DYRK1A as a search molecule.

### Analysis of ΔT_m_ upon Inhibitor Binding

Changes in T_m_ caused by small-molecule binding were correlated for 433 compounds that caused an increase in T_m_ of >2°C for either DYRK1A or DYRK2 and for which a measurement was available for both proteins. The measurements were made according to established protocols (Niesen et al., 2007).

### Autophosphorylation

DYRK1A proteins were mixed with ATP (1 mM) and Mg^2+^ (2 mM) and incubated at room temperature. Western blot analysis of phosphotyrosine was performed using rabbit anti-pTyr antibody (Cell Signaling Technology).

### In-Solution Digestion

The DYRK1A protein from in vitro autophosphorylation was diluted in 100 μl of an 8 M urea buffer (6 M urea, 2 M thiourea in 10 mM HEPES [pH 8]). The protein was reduced for 30 min at room temperature with 1 mM dithiothreitol and then alkylated for 15 min by 5.5 mM iodoacetamide. Endoproteinase Lys-C (Wako) was added 1:100 (w/w) and the lysates were digested for 4 hr at room temperature. The resulting peptide mixtures were diluted 4-fold with deionized water to achieve a final urea concentration below 2 M. Trypsin (modified sequencing grade; Promega) was added 1:100 (w/w) and the sample was digested overnight. Trypsin and Lys-C activity was quenched by acidification of the reaction mixtures with a 10% TFA solution to pH ∼2. The peptide mixture was desalted and concentrated on a reverse-phase C18 StageTip (Rappsilber et al., 2007) and eluted with 2 × 20 μl of 60% acetonitrile in 0.3% TFA.

### In-Gel Digestion

The excised gel plugs with the DYRK1A protein were digested in situ with trypsin as previously described (Shevchenko et al., 2006).

### Phosphopeptide Enrichment and Analysis

Phosphopeptides were enriched using titansphere chromatography as described previously (Olsen et al., 2006), and analyzed by online nanoflow LC-MS/MS as described previously (Olsen et al., 2006) with a few modifications. More detail is available in Supplemental Experimental Procedures.

### Kinase Assays

Peptide substrates were chemically synthesized by Thermo Peptides or Generon. The phosphorylation reactions were measured using a spectrophotometric assay (Adams et al., 1995) in which ADP production is coupled to NADH oxidation by pyruvate kinase and lactate dehydrogenase (LDH). The reaction was followed by the decrease in NADH fluorescence (excitation 350 nm, emission 460 nm). The assay mixture contained 30 U/ml LDH, 12 U/ml pyruvate kinase, 1 mM phosphoenolpyruvate, 0.2 mM NADH, 25 mM HEPES (pH 7.5), 150 mM NaCl, 5 mM MgCl_2_, and DYRK1A (wild-type or mutants). The concentration of the consensus substrate peptide (RARPGTPALRE) was 0.25 mM. After incubation for 1–5 min, the reaction was initiated by the simultaneous addition of 1 mM ATP. The initial reaction rate was used to compare activities of wild-type and mutants. Control reactions in the absence of substrate were used to detect ATPase activity for basal correction. A K188R mutant was also measured to control for any peptide-stimulated ATPase activity.

### Determination of Peptide Phosphorylation Specificity

Peptides derived from HeLa cell-lysate digests that were phosphorylated by DYRKs were identified as in published procedures (Kettenbach et al., 2012).

## Supplemental Information

Document S1. Figures S1–S7 and Supplemental Experimental Procedures
