# Supplementary material for: Structures of Down Syndrome Kinases, DYRKs, Reveal Mechanisms of Kinase Activation and Substrate Recognition
Source: Structure. 2013 Jun 4;21(6):986–96. doi: 10.1016/j.str.2013.03.012 (PMC3677093; doi:10.1016/j.str.2013.03.012)
Supplement: Document S1. Figures S1–S7 and Supplemental Experimental Procedures [file mmc1.pdf]

Structure, Volume *21*

Supplemental Information

Structures of Down Syndrome Kinases, DYRKs,

Reveal Mechanisms of Kinase Activation

and Substrate Recognition

Meera Soundararajan, Annette K. Roos, Pavel Savitsky, Panagis Filippakopoulos, Arminja N. Kettenbach, Jesper V. Olsen, Scott A. Gerber, Jeyanthy Eswaran, Stefan Knapp, and Jonathan M. Elkins

Inventory of Supplemental Information

Figure S1. Sequence alignment of the human DYRK family, related to Figure 1A.

Figure S2. Chemical structure of the inhibitor DJM2005 used in the DYRK1A crystal structure, related to Table 1.

Figure S3. Active site comparison of DYRK1A with inhibitor DJM2005 bound and apo-DYRK2, related to Figure 2C.

Figure S4. Comparison of the DH box region of DYRK1A with the analogous region in CLK3, related to Figure 3A.

Figure S5. Identification of peptides by mass spectrometry from an autophosphorylated DYRK1A sample, related to Table 2.

Figure S6. Kinase activity measurement of wild-type DYRK1A and Y321F mutant, using an ASF derived peptide or the consensus peptide used for crystallisation, related to Figure 5B.

Figure S7. Substrate preference of DYRK2 from analysis of phosphorylation of a library of naturally occurring human peptides produced by proteolysis of cell extracts, related to Figure 6A.

Supplemental Experimental Procedures

Supplementary Figures

Figure S1.

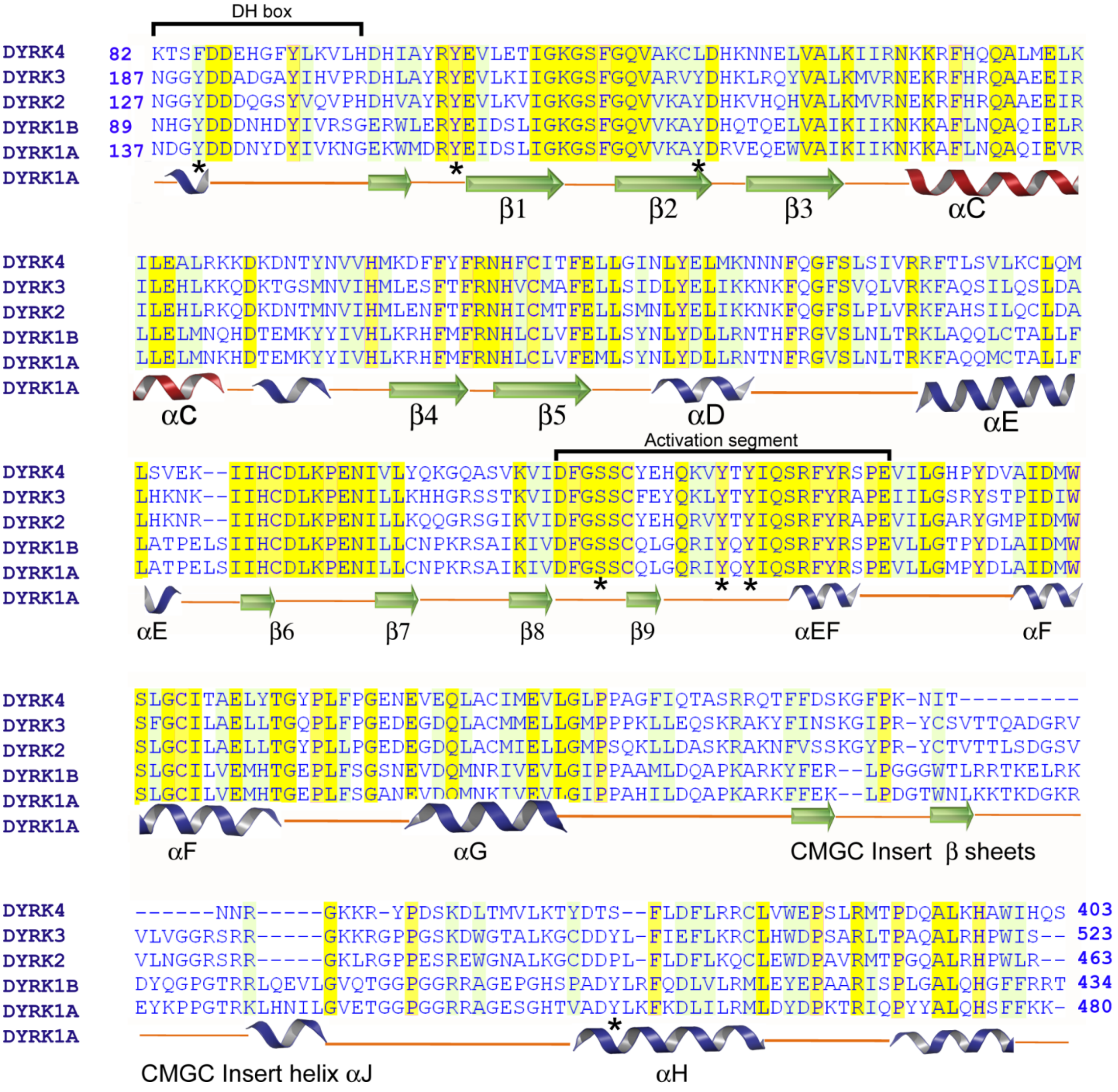

Figure S1, related to Figure 1A. Sequence alignment of the human DYRK family with secondary structure elements shown below the alignement. The main structural elements are labeled and

identified auto-phosphorylation sites are indicated by a *. Residues fully or partially conserved across human DYRKs are indicated by yellow or green shading respectively.

Figure S2

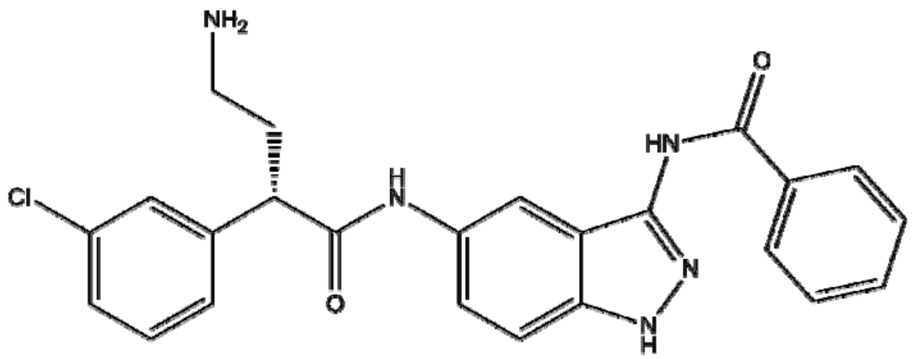

Figure S2, related to Table 1. Chemical structure of the inhibitor DJM2005 used in the DYRK1A crystal structure. Inhibitor kindly provided by the laboratory of Kevan Shokat (UCSF).

Figure S3

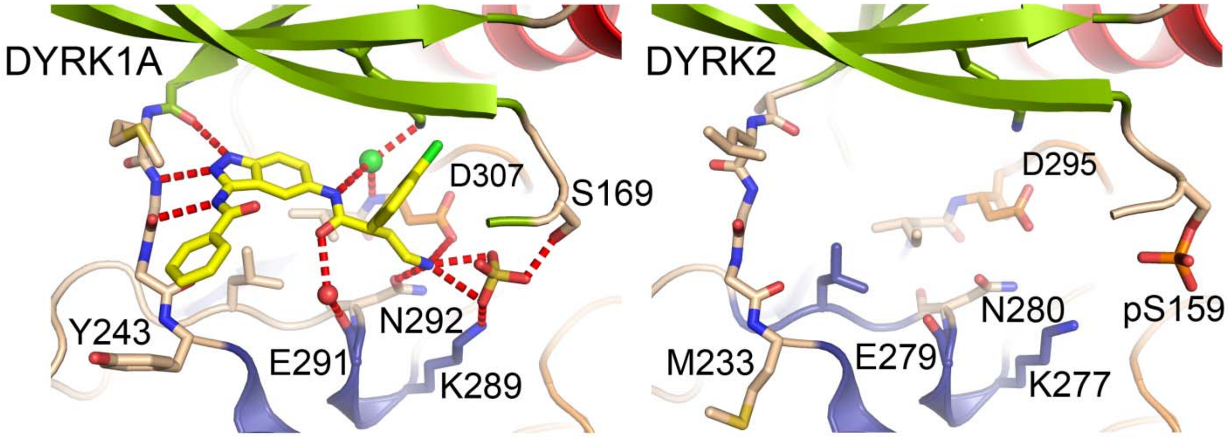

Figure S3, related to Figure 2C. Active site comparison of DYRK1A with inhibitor DJM2005 bound and apo-DYRK2. Part of helix β1 has been removed for clarity. The inhibitor is coloured yellow and hydrogen bonds are shown as dashed red lines. The conservation of residues involved in hydrogen bonding to inhibitor DJM2005 is shown, as is the phosphorylated Ser159 in a similar position to the bound sulphate molecule in DYRK1A.

Figure S4

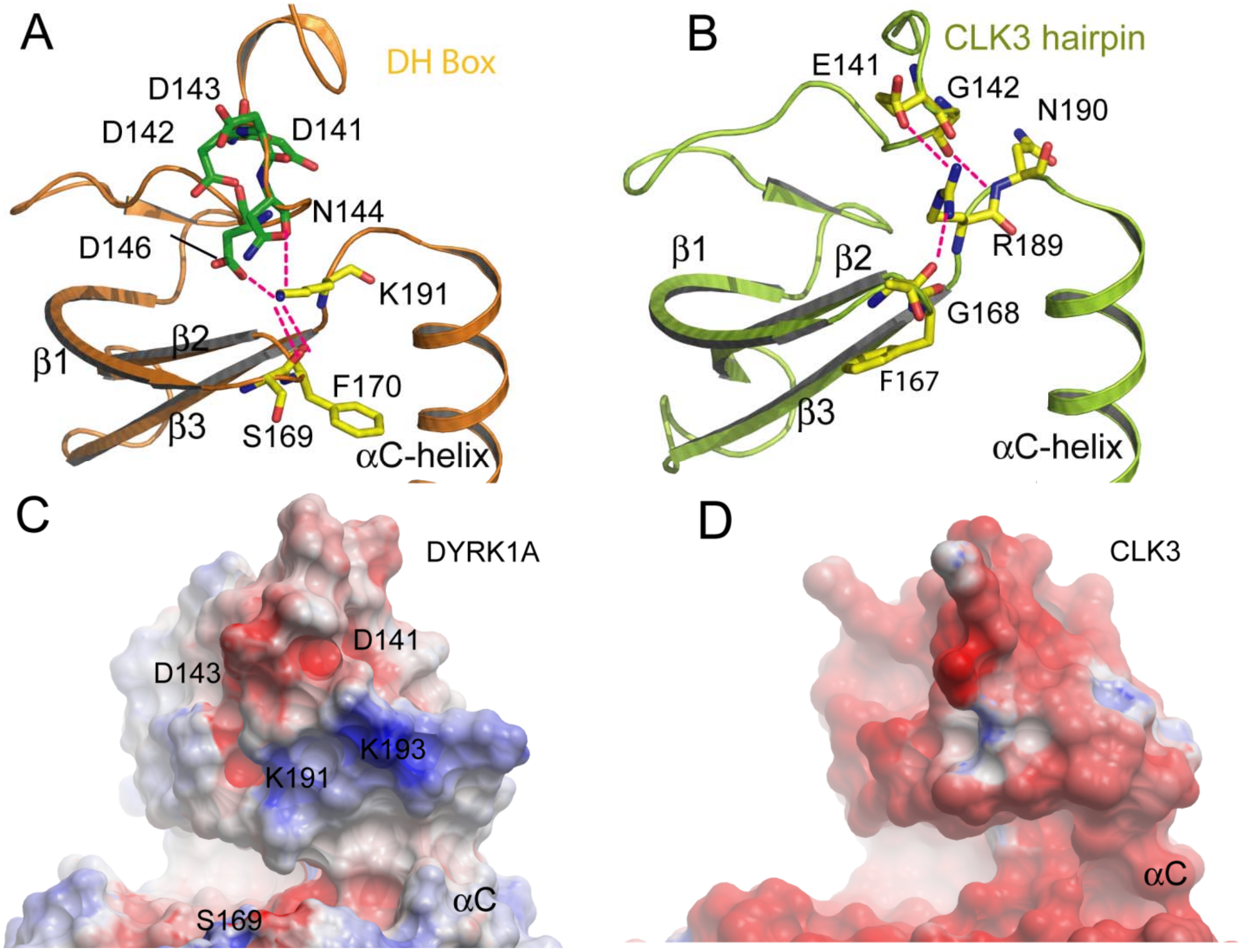

Figure S4, related to Figure 3A. Comparison of the DH box region of DYRK1A with the analogous region in CLK3 showing the conserved tertiary structure but variation in charged surface. **(A)** DYRK1A, **(B)** CLK3, **(C)** DYRK1A surface coloured by charge, **(D)** CLK3 surface coloured by charge.

Figure S5

A

Modified phosphopeptide sequence	m/z	Mass (Da)	Mass error	charge	Mascot Score	PTM score
**AY(ph)DRVEQEWVAIK**	562.9356	1685.7848	0.87	3	7	35.3
**IVDFGS(ph)SCQLGQR**	773.842	1545.6695	1.97	2	31	35.3
**IY(ph)QY(ph)IQSR**	615.7507	1229.4868	-2.34	2	35	114.7
**IYQY(ph)IQSR**	575.7675	1149.5205	-2.57	2	50	150.9
**LPDGT(ph)WNLK**	562.2657	1122.5168	3.79	2	16	36.8
**RAGESGHTVADY(ph)LK**	528.5802	1582.7186	2.01	3	13	19.2
**VYNDGY(ph)DDDNYDYIVK**	1025.9039	2049.7933	1.55	2	21	44.3
**WM(ox)DRY(ph)EIDSLIGK**	574.5931	1720.7576	1.46	3	26	21.1
**WMDRY(ph)EIDSLIGK**	569.2615	1704.7628	1.55	3	20	28.2

B

IY(ph)QY(ph)IQSR, expected m/z = 615.7514 (2+)

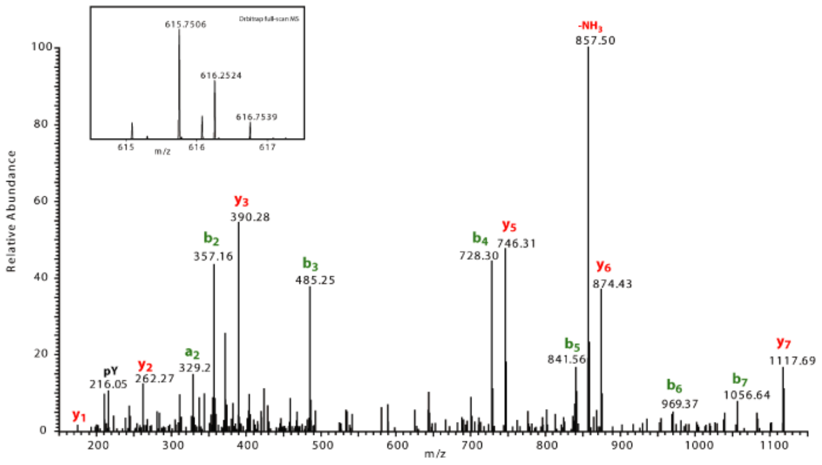

VYNDGY(ph)DDDNYDYIVK, expected m/z = 1025.9013 (2+)

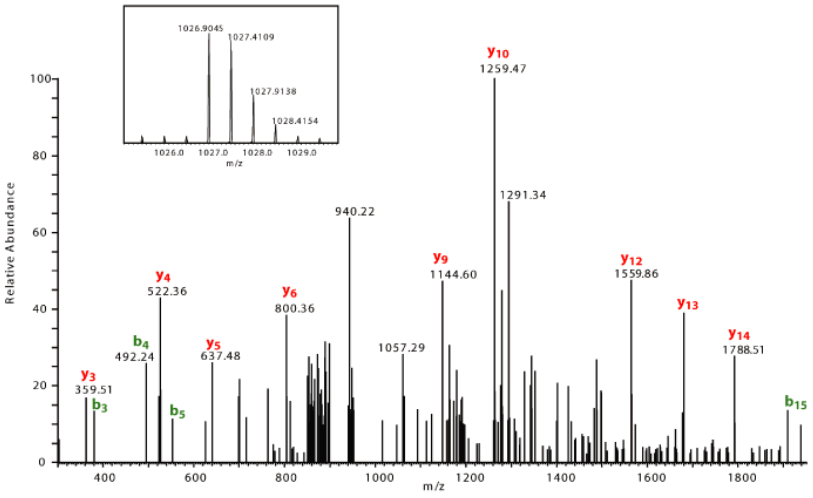

Figure S5, related to Table 2. Identification of peptides by mass spectrometry from an autophosphorylated DYRK1A sample. **(A)** Table showing the phosphorylated peptides identified using LC-MS/MS from phospho enriched samples of purified, autophosphorylated, DYRK1A 127-485. **(B)** Identification of phosphorylation sites of wild type DYRK1A using tryptic digest and phosphopeptide identification by MS/MS. Two representative MS/MS analyses are given including IYQYIQSR from the activation loop and VYNDGYDDDNYDYIVK from the DH box regions.

Figure S6

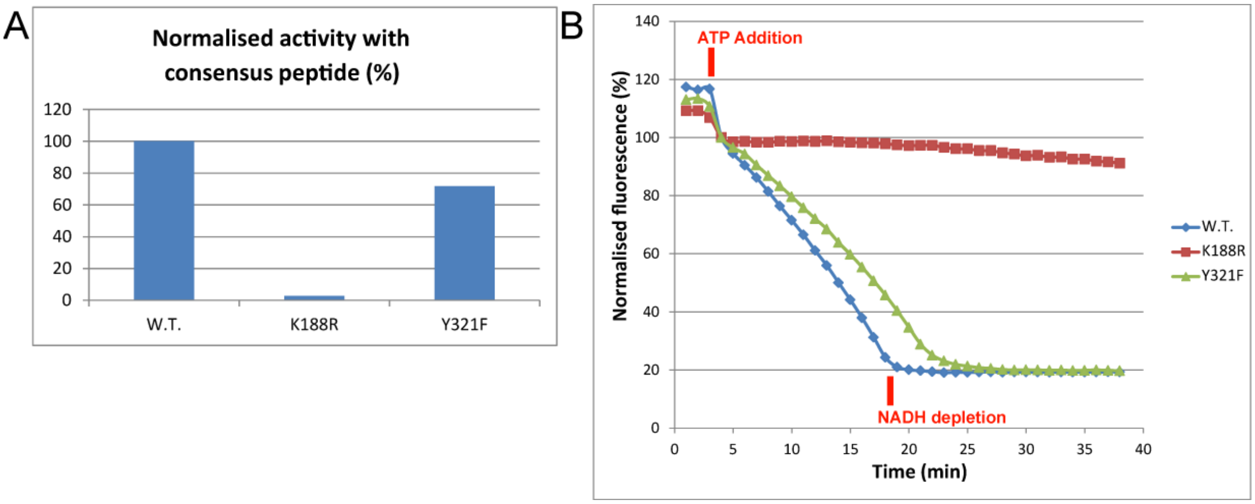

Figure S6, related to Figure 5B. Kinase activity measurement of wild-type DYRK1A and Y321F mutant, using the consensus peptide used for crystallisation. DYRK1A K188R mutant was included as a control. **(A)** Normalised percentage activity. **(B)** Graph of raw kinetic data showing depletion of NADH (normalised) over time. The time points for reaction initiation (by ATP addition) and end point (NADH depletion) are marked. The values in (A) are calculated from the slope gradients in (B) over the region from just after ATP addition to just before NADH depletion.

Figure S7

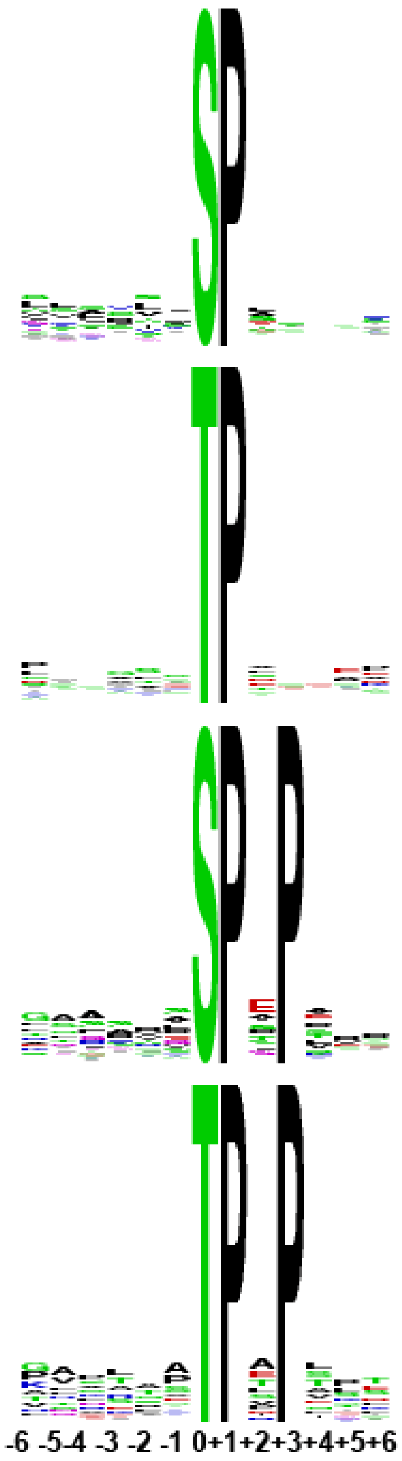

Figure S7, related to Figure 6A. Substrate preference of DYRK2 from analysis of phosphorylation of a library of naturally occurring human peptides produced by proteolysis of cell extracts. The motifs are ordered from most observed to least observed.

Supplemental experimental procedures

Expression and Purification

Clones were transformed into *Escherichia coli* BL21 (DE3) competent cells containing the pRARE2 plasmid from the commercial Rosetta strain, and the transformants were used to inoculate 10 ml of Luria–Bertani (LB) medium containing 50 µg/ml kanamycin and 34 µg/ml chloramphenicol which was incubated overnight at 37 °C. This culture was used to inoculate 1 litre of LB medium with 50 µg/ml kanamycin and grown at 37 °C until an OD600 of 0.5 was reached. Expression was induced with 1 mM IPTG (isopropyl β-D-thiogalactoside) at 18 °C. The cells were harvested by centrifugation, resuspended in lysis buffer (50 mM HEPES pH 7.5, 500 mM NaCl, 5% glycerol, 5 mM imidazole and 0.5mM TCEP) and frozen at -80 °C until further use. The frozen cell pellet was thawed in the presence of protease inhibitors and the cells were lysed using an Emulsiflex C5 high pressure homogenizer (Avestin). The insoluble debris was removed by centrifugation.

The supernatant was bound to Ni-NTA resin (Ni^{2+}-nitrilotriacetate, Qiagen) and washed with 30 column volumes (CV) of lysis buffer and 5 CV of wash buffer (50mM HEPES pH 7.5, 500 mM NaCl, 5% glycerol, 25 mM imidazole and 0.5mM TCEP). The protein was eluted from the resin with 5 CV of elution buffer (50 mM HEPES pH 7.5, 500 mM NaCl, 5% glycerol, up to 250 mM imidazole and 0.5mM TCEP). The eluted proteins were further purified by gel-filtration chromatography using an S200 16/60 column (GE Healthcare) in 25 mM Hepes pH 7.5, 500 mM NaCl, 5 mM DTT. Protein identity was confirmed by mass spectrometry under denaturing conditions (DYRK1A wild-type: expected 40403 Da, observed primarily 40484 Da; DYRK2 expected 49279 Da, observed primarily 49360). For both DYRK1A and DYRK2 the number and range of phosphorylation states was variable between different expressions of each protein.

For co-expression of DYRK1A with lambda-phosphatase, the procedure was as above except that the cell line included a lambda-phosphatase expression plasmid in place of the pRARE2 plasmid.

Crystallization and Data Collection

All crystals were obtained using the sitting drop vapour diffusion method at 4°C.

DYRK1A with inhibitor: The protein (14 mg/ml) was mixed with 1 mM DJM2005. Crystals grew from a mixture of 200 nl of this protein solution and 100 nl of a well solution containing 34% (v/v) PEG 300, 0.1 M Li_2SO_4, 0.1M Tris pH 8.5. Crystals were flash-frozen in liquid nitrogen. Diffraction data were collected from 2 parts of a single 100 µM crystal.

DYRK1A with peptide: The protein (15 mg/ml) was mixed with 1 mM DJM2005 and 1.5 mM substrate peptide (RYRPGTPALRE). Crystals grew from a mixture of 75 nl of this protein solution and 75nl of a well solution containing 0.2 M sodium formate, 20% (w/v) PEG3350, 10% ethylene glycol. Crystals were cryo-protected using the well solution supplemented with 30% ethylene glycol and flash-frozen in liquid nitrogen.

DYRK2: Crystals grew from a mixture of 200 nl protein (7 mg/ml) and 100 nl of a well solution containing 1.26 M $(NH_4)_2SO_4$, 0.2 M Li_2SO_4, 0.1 M Tris pH 8.5. Crystals were cryo-protected using the well solution supplemented with 2M Li_2SO_4 and flash-frozen in liquid nitrogen.

Phosphopeptide enrichment and analysis.

Phosphopeptides were enriched using titansphere chromatography as described (Olsen et al., 2006). Briefly, phosphopeptides extracted from in-gel and in-solution digests were enriched on titanium dioxide beads (10 µm titansphere, GL Sciences, Japan) pre-coated with 2,5-dihydroxybenzoic acid (2,5-DHB). 5 µg of coated-beads were added to each SCX fraction and incubated under rotation for 15 min. The beads were washed once with 200 µL 30% acetonitrile in 3% TFA, and once with 200 µL 60% acetonitrole in 0.3% TFA and transferred in 200 µL 75% acetonitrile in 0.3% TFA on top of a RP-C8 StageTip. The bound phosphopeptides were eluted directly into a 96-well plate by two volumes of 20 µL 25% acetonitrole in 15% NH_4OH-water (pH > 11). The eluates were immediately dried in a speed-vac at 60 °C.

The dried phosphopeptide mixtures were acidified with 5% acetonitrile in 0.3% tri-fluoro acetic acid (TFA) to an end volume of 8 µL and analyzed by online nanoflow liquid chromatography tandem mass spectrometry (LC-MS/MS) as described previously (Olsen et al., 2006) with a few modifications. The peptides were analyzed on an EASY-nLC™ system (Proxeon Biosystems, Odense, Denmark) connected to the LTQ-Orbitrap XL (Thermo Electron, Bremen, Germany) through a nanoelectrospray ion source using a top10 method. Survey full scan MS spectra (from m/z 300 – 2000) were acquired in the orbitrap with resolution R=60K at m/z 400 (after

accumulation to a 'target value' of 1x 10^6 in the linear ion trap). The ten most intense peptide ions with charge states ≥ 2 were sequentially isolated to a target value of 5x 10^3 and fragmented in the linear ion trap by multi-stage activation (MSA or pseudo MS3). All fragment ion spectra were recorded with the LTQ detectors. For all measurements with the orbitrap detector a lock-mass ion from ambient air (m/z 445.120024) was used as an internal calibrant as described (Olsen et al., 2005).

Peptide identification and quantitation by MASCOT and MaxQuant

Raw Orbitrap full-scan MS and ion trap MSA spectra were processed by MaxQuant as described (Cox et al., 2009). Accurate precursor masses were determined using the entire LC elution profiles and MS/MS spectra were merged into peak-list files (*.msm). Phosphopeptides were identified by Mascot (Matrix Science, London, UK) via automated database matching of all tandem mass spectra against an in-house curated, concatenated target/decoy database; a forward and reversed version of the human International Protein Index (IPI) sequence database (version 3.37; 138,632 forward and reversed protein sequences from EBI (http://www.ebi.ac.uk/IPI/)) supplemented with common contaminants. Tandem mass spectra were initially matched with a mass tolerance of 7 ppm on precursor masses and 0.5 Da for fragment ions, and strict trypsin specificity and allowing for up to 3 missed tryptic cleavage sites. Cysteine carbamidomethylation (Cys +57.021464 Da) was searched as a fixed modification, whereas N-acetylation of protein (N-term +42.010565 Da), N-pyro-glutamine (Gln –17.026549), oxidized methionine (+15.994915 Da) and phosphorylation of serine, threonine and tyrosine (Ser/Thr/Tyr +79.966331 Da) were searched as variable modifications.

Peptide filtering and phosphosite localization

The resulting Mascot result files (*.dat) were loaded into the MaxQuant software suite for further processing. In MaxQuant we fixed the estimated false discovery rate (FDR) of all peptide and protein identifications at 1%, by automatically filtering on peptide length, mass error precision estimates and Mascot score of all forward and reversed peptide identifications (Cox and Mann, 2008). Finally, to pinpoint the actual phosphorylated amino acid residue(s) within all identified phosphopeptide sequences in an unbiased fashion, MaxQuant calculated the

localization probabilities of all putative serine, threonine and tyrosine phosphorylation sites using the PTM score algorithm as described (Olsen et al., 2006).

In-gel digestion

The excised gel-plugs with the DYRK1A protein were digested *in situ* with trypsin as previously described (Shevchenko, Tomas, Havlis, Olsen, & Mann, 2006). Briefly, the gel slices were destained by 50% acetonitrile in 25 mM ammonium bicarbonate (ABC) buffer, pH 8.5. Protein disulfides were reduced by treatment with 10 mM DTT for 30 minutes and alkylated by 55 mM IAA for 45 min at RT. The gel-plugs were washed twice with neat acetonitrile and digested with trypsin overnight. The resulting tryptic peptides were extracted by 30% acetonitrile in 3% TFA, reduced in a speed vac and desalted and concentrated on RP-C18 StageTips as described above.

Supplemental References

Cox, J., Matic, I., Hilger, M., Nagaraj, N., Selbach, M., Olsen, J. V., & Mann, M. (2009). A practical guide to the MaxQuant computational platform for SILAC-based quantitative proteomics. *Nature Protocols*, *4*(5), 698–705. doi:10.1038/nprot.2009.36

Olsen, J. V., Blagoev, B., Gnad, F., Macek, B., Kumar, C., Mortensen, P., & Mann, M. (2006). Global, in vivo, and site-specific phosphorylation dynamics in signaling networks. *Cell*, *127*(3), 635–48. doi:10.1016/j.cell.2006.09.026

Olsen, J. V., de Godoy, L. M. F., Li, G., Macek, B., Mortensen, P., Pesch, R., Makarov, A., et al. (2005). Parts per million mass accuracy on an Orbitrap mass spectrometer via lock mass injection into a C-trap. *Mol. Cell. Proteomics*, *4*(12), 2010–21. doi:10.1074/mcp.T500030-MCP200

Shevchenko, A., Tomas, H., Havlis, J., Olsen, J. V., & Mann, M. (2006). In-gel digestion for mass spectrometric characterization of proteins and proteomes. *Nature Protocols*, *1*(6), 2856–60. doi:10.1038/nprot.2006.468
